# Lipid droplets and viruses: a dynamic battlefield of cellular resources

**DOI:** 10.52601/bpr.2026.250053

**Published:** 2026-10-31

**Authors:** Xue Ling, Zifang Zheng, Shuang Qiao, Shuangquan Zhang, Jie Wu, Shuqi Xiao

**Affiliations:** 1 State Key Laboratory of Animal Disease Control and Prevention, College of Veterinary Medicine, Lanzhou University, Lanzhou Veterinary Research Institute, Chinese Academy of Agricultural Sciences, Lanzhou 730000, China; 2 Gansu Province Research Center for Basic Disciplines of Pathogen Biology, Lanzhou Veterinary Research Institute, Chinese Academy of Agricultural Sciences, Lanzhou 730046, China

**Keywords:** LDs, Virus, Hijack, Antiviral, Immunity

## Abstract

Lipid droplets (LDs) are highly conserved, evolutionarily dynamic organelles. In addition to their canonical roles in energy homeostasis, membrane biosynthesis, and lipid signaling, accumulating evidence highlights their critical involvement in viral pathogenesis. As obligate intracellular parasites, viruses are entirely dependent on their host’s cellular machinery. To replicate, they must hijack LDs to obtain essential lipids and energy, which are critical for assembling viral replication complexes and producing new virions. However, host cells do not passively accept viral invasion. Recent evidence demonstrates that pathogen infection triggers the innate immune response of host cells. This response, in turn, rewires lipid metabolism to drive the formation of new LDs. These newly formed LDs are characterized by surfaces enriched with immune proteins. These specialized lipid droplets serve as defensive structures that combat pathogen invasion through both direct and indirect mechanisms. This article provides a systematic review of the dual roles of LDs in viral infection: on the one hand, LDs are hijacked by viruses to facilitate their replication; on the other hand, they serve as central hubs in innate immunity, participating in host cell antiviral defense mechanisms. These findings lend substantial support to the hypothesis that “LDs constitute critical components of innate immunity”, while offering novel perspectives for understanding virus-host interactions. Future investigations should focus on elucidating the specific molecular mechanisms by which LDs mediate immune regulation, and explore innovative antiviral therapeutic strategies through targeted modulation of LD metabolism.

## INTRODUCTION

### Composition of LD

Lipid droplets (LDs) are structurally unique and highly conserved organelles ubiquitously present in both prokaryotes (bacteria) and eukaryotes (yeast, plants, insects, and animal cells). Their structural features are markedly distinct from those of traditional double-membrane-bound organelles, being characterized by a monolayer phospholipid membrane encapsulating a hydrophobic core. This monolayer membrane is composed of distinctive fatty acid species. The neutral lipid core of LDs primarily consists of triacylglycerols (TAG) and cholesterol esters (CE) (Mathiowetz and Olzmann 2024; Peng *et al*. 2025), while other hydrophobic molecules may also reside within this compartment, including acylceramides, squalene, as well as lipophilic drugs and vitamins (Roberts and Olzmann 2020).

The surface of LDs harbors LD-specific proteins that play pivotal roles in lipid metabolism. These LD-specific proteins serve as core molecular regulators of LD homeostasis and functionality (Qin *et al*. 2022), and are classified into two categories based on their subcellular localization and binding modes: Class I proteins (resident) and Class II proteins (recruited). Class I proteins, such as acyl-CoA synthetase long-chain family member 3 (ACSL3), glycerol-3-phosphate acyltransferase 4 (GPAT4), UBX domain-containing protein 8 (UBXD8), lysosomal diacylglycerol acyltransferase (LDAH), and hydroxysteroid 17-beta dehydrogenase 11 (HSD17B11), typically utilize their hydrophobic hairpin domains embedded in the endoplasmic reticulum (ER) to associate with the phospholipid membrane. They are incorporated into nascent LDs via lateral diffusion from the ER through membrane continuity between the ER and maturing LDs (Olarte *et al*. 2022; Song *et al*. 2022). In contrast, Class II proteins, including lysine-specific demethylase 1 (LSD1), cellular gene inducible by IFN-gamma 58 (CGI-58), and chaperonin containing TCP1 subunit 1 (CCT1), primarily anchor to the LD monolayer through amphipathic helices or fatty acid modifications, stabilizing helical structures to retain their localization on LDs (Caillon *et al*. 2020; Choi *et al*. 2023; Olzmann and Carvalho 2019; Roberts and Olzmann 2020). Both classes of LD-specific proteins interact with the monolayer phospholipid membrane via distinctive structural domains, coordinating the regulation of LD metabolic homeostasis and mediating interorganellar communication between LDs and other cellular compartments.

### LD biogenesis

LDs play a pivotal role in maintaining cellular energy storage and lipid homeostasis. LD biogenesis originates from the ER and involves three key steps: neutral lipid synthesis and oil body formation, LD budding, and LD growth/maturation (Olzmann and Carvalho 2019).

Fatty acids (FAs), essential substrates for neutral lipid synthesis, are acquired via two pathways: (1) *de novo* intracellular synthesis, where glucose-derived precursors are enzymatically converted into FAs, and (2) extracellular uptake mediated by FA transport proteins (FATPs) or FA translocases (CD36). Regardless of their origin, FAs undergo esterification and activation by acyl-CoA synthetases (ACS). Subsequent enzymatic cascades involving glycerol-3-phosphate o-acyltransferase (GPAT), 1-acylglycerol-3-phosphate o-acyltransferase (AGPAT), phosphatidic acid phosphatase (PAP)/lipin, and diacylglycerol o-acyltransferase (DGAT) families sequentially esterify diacylglycerol (DG) into TAG (Pol *et al*. 2014). The *de novo* synthesis of CE requires cholesterol molecules generated through a series of catalytic reactions from sterol precursors (Luo *et al*. 2020), followed by esterification via acyl-coA: cholesterol acyltransferase (ACAT) (Zhu *et al*. 2021). When CE and TAG accumulate in the ER beyond a critical threshold, neutral lipids coalesce with membrane phospholipids to form lipid lenses (Renne *et al*. 2020). The progressive accumulation and esterification of lipids drive the lens to expand into the cytosol. This process culminates in the formation of nascent LDs-spherical organelles whose hydrophobic core, enriched in TAGs and CEs, is encapsulated by a monolayer phospholipid membrane (Kassan *et al*. 2013).

Nascent LD formation is governed by diverse biological and biophysical factors, including local ER lipid composition, membrane curvature, and surface tension (Ben M’barek *et al*. 2017; Chorlay *et al*. 2019; Thiam and Ikonen 2021). Beyond neutral lipid-synthesizing enzymes, LD biogenesis requires coordinated actions of specialized proteins (Henne 2023), such as Seipin, PLIN3, and fat storage-induced transmembrane protein 2 (FIT2). Seipin, localized at ER-LD contact sites, acts as an LD nucleation factor (Salo 2023). It facilitates TAG flux into LDs by coupling with LD assembly factor 1 (LDAF1) to form an LDAF1-Seipin complex, enhancing TAG binding. The flexibility of its transmembrane domain enables LD emergence from the ER cytoplasmic leaflet, regulating LD biogenesis and ER-LD metabolite exchange (Arlt *et al*. 2022; Chung *et al*. 2019; Salo *et al*. 2016, 2019; Zoni *et al*. 2021). Notably, Seipin depletion disrupts LD biogenesis and growth but does not abolish it (Wang *et al*. 2016). Perilipins (PLINs), a family of soluble proteins, specifically target LDs by recognizing lipid-packing defects in the monolayer (Giménez-Andrés *et al*. 2021). They stabilize LD surfaces as scaffolding platforms and modulate lipolytic activation of lipases (Kimmel and Sztalryd 2016; Sztalryd and Brasaemle 2017). PLIN3, for instance, binds diacylglycerol-enriched lipids to promote membrane domain formation for LD biogenesis proteins (Khaddaj *et al*. 2023). By recruiting the ER tubule-forming proteins Rtn4 and receptor expression-enhancing protein 5 (REEP5), the ER-resident protein FIT2 helps to either stabilize curvature at growing lipid lenses or facilitate lipid mobilization (Cai *et al*. 2017; Chen *et al*. 2021). Collectively, these proteins orchestrate LD growth and expansion.

LD architecture in the ER is dictated by phospholipid intrinsic curvature. Monolayer lipids with negative intrinsic curvature, such as diacylglycerol (DAG) and phosphatidylethanolamine (PE)-favor LD embedding, while lipids with positive curvature (lysolipids) reduce surface tension to support LD budding (Choudhary *et al*. 2018). Embedded LDs maintain contact with the ER lumen, allowing ER-resident metabolic and signaling proteins to dynamically regulate LD protein and phospholipid composition. Cells exploit a curvature-driven transition, shifting LDs between embedded and emerged states. This dynamic transition allows them to modulate neutral lipid storage homeostasis and LD functionality in response to ER stress or lipid-sensing cues.

### LD degradation

LD catabolism is a central process in intracellular neutral lipid metabolism, driven by two principal pathways: lipolysis and lipophagy to generate free fatty acids (FFAs), glycerol, cholesterol, and other metabolites. These processes are critical for maintaining cellular energy balance, lipid homeostasis, and diverse physiological functions.

Lipolysis is a stepwise enzymatic cascade mediated by three core lipases. Adipose triglyceride lipase (ATGL), the rate-limiting enzyme for TG hydrolysis, comprises an N-terminal patatin domain and a C-terminal LD-anchoring domain. CGI-58 (also known as α/β-hydrolase domain-containing protein 5, ABHD5) acts as a coactivator of ATGL. The interaction between ATGL and PLINs is pivotal for regulating ATGL activity. Under basal conditions, PLIN1/PLIN5 couple ABHD5, inhibiting its binding to ATGL (Granneman *et al*. 2011). However, during prolonged exercise, fasting, or pathogenic stimuli, protein kinase A (PKA)-mediated phosphorylation triggers PLIN1/PLIN5 dissociation from ABHD5, enabling ABHD5 to activate ATGL via protein–protein interactions. This initiates TAG hydrolysis into DAG and FFAs, with concomitant energy release (Kumar *et al*. 2025a; Li *et al*. 2025a; Zimmermann *et al*. 2004). Hormone-sensitive lipase (HSL), a broad-specificity enzyme with the highest activity toward DG and CE, further hydrolyzes DG into monoacylglycerol (MG) and FFAs, releasing additional energy (Schweiger *et al*. 2006). Monoacylglycerol lipase (MGL), the first characterized MG hydrolase (Karlsson *et al*. 1997), cleaves MG into glycerol and FFAs. The liberated glycerol enters gluconeogenesis to support glucose synthesis (Grabner *et al*. 2017; Mathiowetz and Olzmann 2024), while FFAs are partitioned into mitochondrial β-oxidation for energy production, re-esterification into TGs, conversion into bioactive signaling molecules or precursors, or incorporation into membrane lipids (Grabner *et al*. 2021).

Lipophagy, the autophagic degradation of LDs, encompasses two subtypes: macro-lipophagy and micro-lipophagy. Emerging evidence indicates that lipolysis and lipophagy are not discrete processes but interconnected and mutually reinforcing pathways (Zheng *et al*. 2025). Macro-lipophagy involves selective recognition, engulfment, and lysosomal delivery of entire LDs or LD fragments for TG breakdown into glycerol and FFAs, facilitating energy mobilization and metabolic regulation (Hsia *et al*. 2024; Kumar *et al*. 2025b). This pathway preferentially targets smaller LDs, reflecting a size-dependent constraint in autophagosome engulfment (Schott *et al*. 2019). For larger LDs, cells employ a synergistic degradation network combining chaperone-mediated autophagy (CMA) and macro-lipophagy for progressive catabolism.

The macrolipophagy process comprises sequential stages: selective LD recognition and tagging for sequestration, pre-autophagosomal assembly, phagophore membrane expansion to encapsulate LDs, vesicle elongation facilitating LD envelopment, autophagosome closure via membrane nucleation, autophagosome-lysosome fusion through soluble N-ethylmaleimide-sensitive factor attachment protein receptor (SNARE) machinery, and terminal LD catabolism mediated by lysosomal hydrolases (Hsia *et al*. 2024). Ubiquitinated LD surface proteins (*e*.*g*., perilipins) recruit autophagy receptors including sequestosome 1 (SQSTM1/p62), neighbor of BRCA1 gene 1 (NBR1), and optineurin (OPTN) (Ding *et al*. 2025), which anchor phagophore membranes by binding microtubule-associated protein 1 light chain 3 (LC3) on autophagosomal membranes (Shao *et al*. 2025). This receptor-mediated targeting directs substrates to autophagosomes, which then dock and fuse with lysosomes via SNARE complexes. Within the resulting autolysosomes, lysosomal acid hydrolases execute the final LD breakdown (Amari *et al*. 2024). Since 2020, studies across hepatocytes, cardiomyocytes, and other cell types have revealed that micro-lipophagy, previously characterized in yeast, plants, and algae, also operates in animal cells (Jauh *et al*. 1999; Ke *et al*. 2024; Schulze *et al*. 2020; Tarique *et al*. 2019; van Zutphen *et al*. 2014; Zhang *et al*. 2020). Micro-lipophagy mediates direct LD uptake through invagination or protrusion of vacuolar/lysosomal membranes. However, the detailed mechanisms underlying micro-lipophagy in mammalian systems remain elusive.

### LD stores nutrients

LDs function as dynamic metabolic sensors within cells, leveraging their lipid compositional plasticity to precisely detect and adapt to fluctuations in nutrient availability. This capability enables them to orchestrate cellular energy allocation and storage strategies, resulting in cell type-specific variations in LD size, abundance, subcellular localization, and composition (Thiam and Beller 2017). The core functionality of LDs stems from their unique neutral lipid core, predominantly composed of TAGs and CEs. TAGs, the most ubiquitous energy storage molecules in biology, are formed by esterification of glycerol with three fatty acids. Their high reduction state and hydrophobicity render them an optimal form for energy storage efficiency (Pan *et al*. 2024). TAG biosynthesis is mediated by two principal pathways: the Kennedy pathway (dominant in eukaryotes) and the acyl-CoA-independent pathway (operating in plants, algae, and fungi) (Gong *et al*. 2023). LD biogenesis is not a passive lipid aggregation process but a tightly regulated biological event. During formation, LDs recruit specialized proteins (*e*.*g*., PLIN2, PLIN3, ATGL, and RAB18) to their surfaces, establishing functionally specialized metabolic platforms (Herrera-Moro Huitron *et al*. 2023). The dynamic equilibrium between LD synthesis and degradation underpins cellular metabolic homeostasis. Under nutrient-rich conditions, LDs expand their storage capacity via activation of transcription factors such as sterol regulatory element binding protein (SREBP) and peroxisome proliferator-activated receptor gamma (PPARγ). Conversely, during fasting or energy stress, LDs mobilize stored lipids through two synergistic pathways: lipolysis (mediated by the enzymatic triad ATGL, HSL, and MGL) (Haemmerle *et al*. 2006) and lipophagy (Bosch *et al*. 2020a), thereby fueling cellular energy demands. This dual-engine degradation strategy optimizes spatiotemporal energy release kinetics-acute demands are met via rapid lipolysis, while chronic energy stress engages lipophagy. Moreover, the bifurcated metabolic fate of FFAs-β-oxidation for energy production versus membrane lipid synthesis achieves a precise balance between immediate energy supply and cellular repair mechanisms.

## VIRUSES HIJACK LD TO FACILITATE THEIR REPLICATION

Viruses, as obligate intracellular pathogens, intricately rewire host lipid metabolic networks to sustain their life cycles. LDs, central hubs for cellular lipid storage and metabolism, play regulatory roles across viral invasion, assembly, proliferation, and release. By dynamically interacting with organelles such as the ER, Golgi apparatus, mitochondria, and peroxisomes, LDs establish “metabolic highways” for lipid exchange, participating in synthesis, storage, degradation, and recycling pathways (Li *et al*. 2025b). Therefore, viruses frequently hijack and exploit LDs to spatiotemporally regulate multiple phases of their life cycle through precise molecular control. The virus establishes viral replication compartments (RCs) by remodeling host membrane systems and hijacking the LD metabolic network, thereby orchestrating the targeted delivery of energy substrates and membrane constituents. This process ultimately forms the “LD-virus interaction axis” to facilitate viral invasion, replication, assembly, and immune evasion, enabling sophisticated circumvention of host defense mechanisms through coordinated redirection of cellular resources.

### Positive-strand RNA viruses manipulate LD

LDs support viral membrane remodeling and replication compartment formation. Severe acute respiratory syndrome coronavirus 2 (SARS-CoV-2) relies on cholesterol released from LDs for host membrane fusion during entry (Bolland *et al*. 2025). Viral infection activates the transcription factor SREBP to induce lipid remodeling and enhance LD biogenesis (Dias *et al*. 2020; Soares *et al*. 2023). Enhanced LD-ER and LD-mitochondria coupling promotes coordinated LD synthesis and lipolysis, providing energy substrates and membrane precursors for viral replication (Yue *et al*. 2023). Hepatitis C virus (HCV) upregulates lipin1, a key enzyme in glycerophospholipid biosynthesis, to facilitate viral envelope formation (Mingorance *et al*. 2018). It degrades apolipoprotein B (ApoB) via non-ubiquitin pathways to promote lipid accumulation (Wang *et al*. 2021), and activates nuclear factor kappa B (NF-κB) and cellular myelocytomatosis oncogene (c-Myc) activation upregulates phospholipase A2 group 4C (PLA2G4C), inducing dissociation of lipolytic factors from LD surfaces and promoting LD stabilization/expansion via intra-LD lipid retention (Ito *et al*. 2025). The HCV NS5A and core proteins cooperatively recruit DGAT1, subsequently co-localizing onto LDs generated through DGAT1 enzymatic activity to establish viral assembly platforms (Camus *et al*. 2024; Chen and Harris 2023; Reichert *et al*. 2024). Furthermore, α/β hydrolase domain-containing protein 5 (ABHD5) synergizes with ATGL to utilize TG hydrolysis for production and assembly of infectious HCV particles (Vieyres *et al*. 2020).

For *Flaviviridae* viruses, LDs serve as strategic platforms for viral protein recruitment and assembly complex formation, ultimately enhancing virion production (Yang *et al*. 2023). Dengue virus (DENV) recruits fatty acid synthase (FASN) to replication sites via NS3 interactions, augmenting fatty acid synthesis (Heaton *et al*. 2010), and effectively promoting LD accumulation (Samsa *et al*. 2009). These LDs are subsequently exploited as viral assembly scaffolds to facilitate particle morphogenesis. Concurrently, DENV infection induces lipophagy-mediated FFA mobilization to mitochondria for β-oxidation-driven ATP synthesis, fueling viral propagation (Heaton and Randall 2010). Zika virus (ZIKV) employs its NS2B protease to recruit host diacylglycerol acyltransferase 2 (DGAT2) into viral replication compartments (Luo *et al*. 2024). Within these compartments, the virus upregulates lipogenic proteins and suppresses lipolytic enzymes. This metabolic rewiring drives LD biogenesis and significantly expands neuronal LD pools, thereby enhancing viral replication (Dias *et al*. 2023). Japanese encephalitis virus (JEV) capsid protein co-localizes with LDs to potentiate virion egress (Ishida *et al*. 2019). Classical swine fever virus (CSFV) orchestrates FASN recruitment to ER-NS4B complexes, inducing fatty acid overproduction and LD expansion. Pharmacological inhibition of fatty acid synthesis pathways significantly impairs CSFV replication, demonstrating FASN-dependent viral biosynthesis (Liu *et al*. 2021). Viruses precisely regulate LD homeostasis to reprogram host metabolic networks. Beyond inducing LD accumulation, flavivirus infection mobilizes the LD-associated protein ancient ubiquitin protein 1 (Aup1) with its cofactor Ube2g2 to trigger lipophagy (Ke 2018; Lan *et al*. 2023). This process serves two critical purposes: it provides membrane precursors for ER-associated viral biogenesis and progeny secretion, and it facilitates the formation of replication organelles and viral assembly. Through a strategy of “bipolar hijacking”, viruses not only co-opt LD biogenesis for lipid storage but also commandeer LD degradation to obtain energy and membranes. This coordinated effort thereby showcases their evolutionary optimization of host lipid metabolism.

Enteroviruses strategically recruit LDs and induce lipolytic degradation to complete their life cycles. Poliovirus (PV) infection leverages the amphipathic helices of 2B/2C proteins to target LDs, facilitating RC recruitment of LDs. These LDs undergo lipolysis to energize RC biogenesis, thereby enhancing virion production (Laufman *et al*. 2019). Enterovirus 71 (EV71) activates the VPS34-PI3P-DFCP1 axis to drive viral-LD coalescence, promoting formation of replication organelles (ROs) essential for productive infection (Wu *et al*. 2024). Both coxsackievirus B3 (CVB3) and EV71 exhibit replication impairment upon pharmacological inhibition of HSL, a key lipolytic enzyme (Belov and van Kuppeveld 2019), demonstrating that enteroviral replication universally exploits RC-anchored lipolysis rather than lipophagy for propagation.

Porcine reproductive and respiratory syndrome virus (PRRSV) manipulates the dynamic remodeling of LDs to reprogram lipid homeostasis, thereby coordinating viral replication demands with host countermeasures. PRRSV upregulates sphingomyelin phosphodiesterase acid-like 3B (SMPDL3B) to suppress lipogenesis and promote lipolysis-mediated LD depletion (Shen *et al*. 2023), while downregulating N-myc downstream-regulated gene 1 (NDRG1) to activate lipophagy-induced LD catabolism (Wang *et al*. 2019). Concurrently, PRRSV activates the canonical RIG-I-MAVS-NF-κB axis to upregulate ras-related protein rab-18 (RAB18), enhancing chaperone-mediated autophagy (CMA)-driven LD breakdown into FFAs. These FFAs undergo mitochondrial β-oxidation to fuel ATP production, energizing viral replication and morphogenesis (Li *et al*. 2024). Notably, Zheng *et al*. found that cells induce LD synthesis by regulating transcription factor YY1 upon sensing PRRSV infection, thereby exerting antiviral effects (Zheng *et al*. 2024). However, studies have revealed that FFAs derived from lipid metabolism are critical for PRRSV replication (Shen *et al*. 2023; Wang *et al*. 2019). If increased LD synthesis is actively induced by PRRSV infection, the virus may directly hijack fatty acids-raw materials for LD synthesis, for replication, bypassing the indirect “LD generation followed by lipophagy” pathway. This suggests that hosts may counteract PRRSV replication by enhancing LD synthesis to sequester FFAs, while PRRSV has evolutionarily acquired counteracting mechanisms to dismantle LDs and release FFAs for its replication. This likely represents the dynamic interplay of exploitation and antagonism underlying PRRSV-LD interactions. Current research predominantly focuses on viral mechanisms to hijack and utilize LDs from diverse perspectives. Future studies should prioritize elucidating host-driven mechanisms of augmented LD synthesis post-infection and corresponding antiviral pathways to fully unravel the complex regulatory networks governing virus-LD dynamics.

### Other viruses manipulate LD

DNA viruses: Marek’s disease virus (MDV) orchestrates *de novo* fatty acid synthesis and prostaglandin E2 (PGE2) production to drive neutral LD accumulation, which is beneficial for viral replication (Boodhoo *et al*. 2019). Epstein-Barr virus (EBV) upregulates FASN expression in B cells, inducing LD biogenesis (Hulse *et al*. 2021). Hepatitis B virus (HBV) induces incomplete autophagy while suppressing lipolysis, resulting in LD retention that potentiates viral replication (Wang *et al*. 2023). Double-stranded RNA viruses: Rotavirus (RVA) establishes viroplasm-LD co-localization, where LD biogenesis becomes essential for replication. Inhibiting or disrupting the synthesis of LDs will reduce the assembly of viroplasm and the viral load (Criglar *et al*. 2020). Negative-strand RNA viruses: rabies virus (RABV) upregulates NDRG1 to activate DGAT1-mediated TAG synthesis, driving LD expansion. The RABV matrix (M) and glycoprotein (G) interact with LD surfaces to facilitate viral budding (Wang *et al*. 2024; Zhao *et al*. 2022). This LD biogenesis concurrently counteracts arachidonic acid (AA)-induced ferroptosis, sustaining host cell viability for viral production (Zhao *et al*. 2024). HCV, DENV, SARS-CoV-2, and ZIKV universally elevate SREBP2 levels to coordinate LD biogenesis. Influenza A virus (IAV) similarly activates SREBP2 to upregulate cholesterol biosynthesis effectors, inducing LD formation that enhances IAV replication efficiency (Li *et al*. 2025c).

## LD ORCHESTRATE VIRAL PATHOGENESIS

LDs orchestrate cellular lipid metabolism and membrane trafficking dynamics, executing critical cytoprotective functions including lipidotoxicity alleviation, inflammatory response modulation, and oxidative stress mitigation (Chen *et al*. 2025). Functioning as a dual nexus for lipid homeostasis and immunological surveillance, LDs constitute the central “metabolic-immune rheostat” governing virus-host battlefield equilibria. Viral hijacking strategies (context-dependent modulation of LD stores) epitomize evolutionary adaptations for resource exploitation and immune evasion. Crucially, the host counteracts this metabolic subversion through multidimensional defense architectures rather than passive surrender as a metabolic hostage.

### LD metabolism modulates viral pathogenesis

LDs emerge as pivotal organelles in innate antiviral immunity, remodeling lipid metabolic networks during pathogen invasion to enact a dual-pronged defense paradigm of “resource sequestration-signal amplification”. Core mechanisms involve: (1) cholesterol biosynthesis downregulation coupled with enhanced influx and restricted efflux, (2) augmented *de novo* fatty acid synthesis and uptake, and (3) strategic modulation of LD-associated enzymatic machinery. This immune-metabolic architecture establishes a tripartite coordinated defense against viral propagation. It integrates immune-driven LD biogenesis, tight coupling between cholesterol metabolism and IFN signaling, and oxysterol-mediated antiviral effects. Together, these components form a multilayered defensive network.

Pathogen infection activates innate immune signaling, driving hypoxia-inducible factor-1 alpha (HIF-1α)-mediated transcriptional reprogramming to upregulate DGAT1/DGAT2 expression and induce LD biogenesis (Knight *et al*. 2018). This process exhibits significant positive correlation with type I/III IFN production. Early infection phases of herpes simplex virus-1 (HSV-1), IAV, DENV, ZIKV, and viral mimetics consistently trigger transient LD accumulation, establishing an “LD-IFN feedforward circuit” (Monson *et al*. 2021a, b). Such metabolic-immune synergy executes critical antiviral functions through dual LD roles: serving as both lipid reservoirs and immunological signalosomes that spatially amplify interferon-stimulated gene (ISG) cascades to curtail viral replication.

Attenuating lipid synthesis restricts substrate availability for invading pathogens. Cholesterol, as a critical modulator of membrane architecture, embeds within phospholipid bilayers to maintain structural integrity and fluidity. Its metabolic reprogramming exerts biphasic regulation in antiviral defense. IFN-I signaling downregulates HMG-CoA reductase (HMGCR), the rate-limiting enzyme in cholesterol biosynthesis, while reduced cholesterol flux reciprocally amplifies type I IFN responses through autocrine/paracrine circuits that potentiate cellular antiviral states (York *et al*. 2015). The ER-resident protein STING mediates cholesterol-sensing antiviral mechanisms: ER cholesterol depletion activates the cGAS-STING-TBK1-IRF3 axis to drive type I IFN production, establishing a self-reinforcing “low cholesterol-STING activation-IFN surge” positive feedback loop (O’Neill 2015).

Sterol 25-hydroxylase (CH25H), an ISG, catalyzes cholesterol conversion to 25-hydroxycholesterol (25HC)-a pleiotropic lipid mediator demonstrating both antimicrobial (Abrams *et al*. 2020) and broad-spectrum antiviral activities. Mechanistically, CH25H exerts direct membrane interference via steric hindrance to inhibit envelope-host membrane fusion, effectively blocking cellular entry of enveloped viruses including PRRSV, SARS-CoV-2, pseudorabies virus (PRV), and bovine parainfluenza virus type 3 (BPIV3) (Ke *et al*. 2017; Lv *et al*. 2019; Wang *et al*. 2017; Zang *et al*. 2020). As a cholesterol derivative, 25HC exhibits broad-spectrum antiviral activity through multifaceted mechanisms. 25HC activates ACAT to deplete membrane cholesterol, thereby restricting coronavirus fusion at the plasma membrane (Wang *et al*. 2020). By suppressing transcription factor EB (TFEB) expression and nuclear translocation, this oxysterol dysregulates the mTOR complex 1 (mTORC1) pathway. This intervention reverses mTORC1 target engagement, thereby blocking its dependent lipophagy and mediating cholesterol metabolic reprogramming. Consequently, stabilized LDs sequester replication-essential lipids while inhibiting transforming growth factor-β1 (TGF-β1) signaling (Zhang *et al*. 2022), ultimately suppressing porcine delta coronavirus (PDCoV) replication and egress (Zhang *et al*. 2025). Nuclear receptor LXR activation enhances 25HC biosynthesis via IFN-γ induction, establishing an antiviral feedforward loop that inhibits HSV-1 infection (Liu *et al*. 2018). Furthermore, 25HC competitively binds oxysterol-binding protein 1 (OSBP1), disrupting sterol extraction from the ER and subsequent transport to viral replication compartments, effectively blocking human rhinovirus (HRV) propagation (Roulin *et al*. 2014). Additionally, 25HC triggers the integrated stress response (ISR) through eIF2α phosphorylation, activating innate immunity while suppressing viral protein translation machinery (Shibata *et al*. 2013).

The essence of viral regulation through lipid droplet metabolism resides in the host’s tripartite strategic paradigm of “lipid sequestration-immune potentiation-stress surveillance”, which converts metabolic vulnerability into defensive supremacy through dynamic resource reallocation. This research frontier establishes an unprecedented framework for metabolically-engineered immunotherapeutics, ranging from cholesterol flux rewiring to oxysterol-mediated antiviral effects and IFN-metabolic circuits. This framework pioneers the strategy of exploiting host lipid circuitry as precision antiviral countermeasures.

### LD mediates immunoregulatory control of viral pathogenesis

Eukaryotic organisms initiate innate immune responses through pattern recognition receptors (PRRs) that precisely detect pathogen-associated molecular patterns (PAMPs) (*e*.*g*., microbial proteins, lipids, and nucleic acids) or Damage-Associated Molecular Patterns (DAMPs, molecular motifs on damaged cells). PRRs are classified into two categories based on subcellular localization: membrane-bound receptors, including Toll-like receptors (TLRs) and C-type lectin receptors (CLRs), and intracellular receptors such as NOD-like receptors (NLRs), retinoic acid-inducible gene-I (RIG-I)-like receptors (RLRs), and DNA sensors (Ge and Ding 2022; Wu *et al*. 2022). Upon PRR activation by PAMPs/DAMPs, signaling cascades are propagated via adaptor proteins, leading to the activation of cytosolic transcription factors, including interferon regulatory factors 3/7 (IRF3/IRF7), activator protein 1 (AP-1), and NF-κB. These factors translocate to the nucleus to drive transcriptional upregulation of proinflammatory cytokines, IFNs, and immune signaling molecules (Bosch and Pol 2022). Crucially, this canonical immune response exhibits deep coupling with LD dynamics: LD accumulation is not a passive pathological hallmark but an actively orchestrated antiviral defense barrier constructed by the host.

LDs exhibit unique “metabolic-immune crosstalk” in antimicrobial immunity by sensing PAMPs and DAMPs to recruit antimicrobial proteins of the innate immune system (Bosch *et al*. 2020b). Through intricate interactions with immune cells such as macrophages and lymphocytes, LDs dynamically fine-tune cellular functionality to combat invading pathogens (Tan *et al*. 2024). Functioning as host-driven innate immune sentinels, LDs demonstrate defensive accumulation even in uninfected bystander cells near pathogen-invaded regions (Bosch and Pol 2022; Sánchez-Álvarez *et al*. 2022). Beyond their role in innate immunity-modulating proinflammatory cytokine production and IFN-I responses-LDs critically engage in adaptive immunity. They facilitate cross-presentation by dendritic cells during viral infection, enabling antigen-specific activation of CD8^+^ T cells (Bougnères *et al*. 2009).

LDs function as dynamic FA reservoirs that actively regulate macrophage polarization by modulating FFA catabolism through mitochondrial respiration, driving the differentiation of bone marrow-derived myeloid cells into immunosuppressive M2-like phenotypes (Wu *et al*. 2019). LDs critically fine-tune immune cell activation, where IFNγ-driven Hypoxia-Inducible Factor-1 Alpha (HIF-1α)-dependent signaling induces LD biogenesis in macrophages. These LDs orchestrate inflammatory responses via eicosanoid production-a process essential for inflammation modulation, innate immunity, and cellular proliferation (Jung *et al*. 2024; Knight *et al*. 2018). Exogenously acquired FAs are transiently stored in LDs and converted into phospholipids, fueling robust proliferation of type 2 innate lymphoid cells (ILC2s) (Karagiannis *et al*. 2020). Notably, macrophage LDs serve as pharmacological reservoirs for lipophilic antibiotics. During Mycobacterium tuberculosis (Mtb) infection, the high lipophilicity of anti-tubercular drug bedaquiline (BDQ) enables its selective accumulation within macrophage LDs. This LD-mediated storage and targeted delivery system potentiates BDQ efficacy by facilitating direct antibiotic transfer to intracellular Mtb reservoirs (Greenwood *et al*. 2019).

### LD-associated proteins orchestrate viral pathogenesis

The LD surface accommodates over 150 functionally diverse proteins, many of which, including lipolytic enzymes and membrane contact site regulators, play pivotal roles in lipid metabolism. Beyond their role as metabolic hubs, LDs also function as strategic platforms for viral replication. For instance, viral proteins like HCV NS5A and DENV NS3 colocalize with LDs or LD-associated proteins to establish viral replication complexes. Recent studies highlight the critical involvement of LD homeostasis and the dynamic regulation of surface proteins in innate immunity. Below, we focus on three extensively studied LD-resident immune proteins: viperin (also known as RSAD2 or CIG5), IFN-inducible GTPases, and histones.

Localizing to lipid droplets via its N-terminal amphipathic α-helix, the interferon-inducible protein viperin is an LD-anchored host factor with broad antiviral activity (Hinson and Cresswell 2009). Widely expressed across diverse cell types, viperin induces the production of IFNs, interferon-stimulated genes (ISGs), and cytokines while regulating cellular metabolism (Jiang and Chen 2011; Saitoh *et al*. 2011; Seo and Cresswell 2013). It exhibits broad-spectrum antiviral activity against DNA viruses, RNA viruses, and retroviruses (Ghosh and Marsh 2020), employing multifaceted mechanisms to execute its antiviral functions.

Viperin exerts metabolic interference by leveraging its radical SAM domain to catalyze the conversion of CTP into 3′-deoxy-3′,4′-didehydro-CTP (ddhCTP). This nucleotide analog acts as a chain-terminating substrate for viral RNA-dependent RNA polymerases (RdRps), thereby suppressing viral translation (Parise *et al*. 2022; Shomar *et al*. 2024). Supporting this mechanism, mutagenesis of two cysteine residues that coordinate the [4Fe-4S] cluster in the SAM domain attenuates viperin’s antiviral efficacy against flaviviruses (Rivera-Serrano *et al*. 2020). Viperin employs distinct mechanisms to inhibit different viruses. It targets farnesyl pyrophosphate synthase (FPPS), an enzyme critical for synthesizing isoprenoid-derived lipids, thereby destabilizing lipid raft integrity and blocking influenza virion release (Wang *et al*. 2007). Separately, its suppression of cholesterol biosynthesis restricts avian infectious bronchitis virus (IBV) replication (Zhang *et al*. 2024). Viperin synergistically amplifies immune signaling to constrain viral infection through distinct pathways. First, it promotes TBK1 ubiquitination and enhances IFN-I signaling through STING interaction, thereby inhibiting HBV replication (Crosse *et al*. 2021). Separately, it activates the GCN2 arm of the MAPK/ZAK-mediated integrated stress response (ISR), which is triggered by ddhCTP-induced ribosomal collisions, to suppress protein translation in West Nile virus (WNV) and ZIKV infections (Hsu *et al*. 2022). Direct viral protein targeting further underpins viperin’s broad antiviral activity. Its C-terminal domain interacts with the DENV-2 capsid (CA) and NS3 proteins to impede early viral RNA synthesis (Helbig *et al*. 2013). It also disrupts HCV NS5A-mediated RC assembly at LD interfaces (Helbig *et al*. 2011). Furthermore, it interferes with the nucleocapsid (N) protein of caprine parainfluenza virus 3 (CPIV3) to inhibit replication (Li *et al*. 2020).

The IFN-inducible GTPase immunity-related GTPase family M member (IRGM) is a lipid droplet-associated autophagy regulator. It directly interacts with viral proteins across five major RNA virus families, including *Paramyxoviridae* and *Flaviviridae*. Notably, IRGM exhibits a dual functional polarity in viral regulation (Grégoire *et al*. 2011). IRGM drives persistent autophagy-dependent replication of peste des petits ruminants virus (PPRV) via the IRGM-HSPA1A axis, where its knockdown significantly impairs PPRV-induced secondary autophagy waves and virion production (Yang *et al*. 2020). Multiple viruses leverage IRGM-mediated autophagy to modulate their replication (Grégoire *et al*. 2011; Petkova *et al*. 2013). However, the outcome is virus-specific: while IRGM interaction with measles virus (MeV) C and HCV NS3 proteins induces proviral autophagy, its engagement with HIV-1 Nef paradoxically suppresses replication via the same pathway. Separately, IRGM functions as a basal negative regulator of IFN signaling, constraining responses by suppressing the cGAS-STING, RIG-I-MAVS, and TLR3 pathways (Jena *et al*. 2020). IRGM deficiency triggers a potent multi-pronged antiviral state. It activates key restriction factors (*e*.*g*., IFITMs, APOBECs), enhances MHC-I antigen presentation, and amplifies stress granule signaling. This coordinated response collectively exerts broad-spectrum inhibition against a wide range of DNA and RNA viruses, including HSV-1, ZIKV, WNV, chikungunya virus (CHIKV), vesicular stomatitis virus (VSV), JEV, and SARS-CoV-2 (Nath *et al*. 2021).

Histones, as LD-resident proteins, exhibit unique immunoregulatory functions in both *Drosophila* and mammals. Histones localized to LDs in *Drosophila* embryos demonstrate potent antimicrobial activity both *in vivo* and *in vitro* (Anand *et al*. 2012). Mechanistically, histones H2B, H2A, and H2AV are anchored to LDs via the Jabba receptor (Stephenson *et al*. 2021). The physiological importance of this pathway is underscored by the finding that Jabba-deficient embryos display heightened susceptibility to bacterial infection (Kolter 2012). Arginine-rich histones H3 and H4 exhibit superior viral neutralization potency compared to lysine-enriched H2A and H2B (Hoeksema *et al*. 2015), with H2A, H2B, H3, and H4 selectively binding influenza A/B matrix protein M1 (Zhirnov and Klenk 1997). Histone H4, in particular, aggregates with influenza A virus (IAV) within neutrophil extracellular traps (NETs), enhancing viral uptake by neutrophils to trigger respiratory bursts that amplify inflammation and compromise viral integrity (Hsieh *et al*. 2021). Histone H1 inhibits human papillomavirus type 11 (HPV-11) replication by directly binding to its DNA helicase E1 (Swindle and Engler 1998). In a distinct and more complex mechanism, histone H2B suppresses CSFV replication by interacting with viral NS4A; this interaction involves H2B K16 lactylation, which activates NF-κB signaling through karyopherin alpha 2 (KPNA2)-mediated p65 nuclear translocation, ultimately inducing IFN-λ expression (Zhu *et al*. 2025). Extracellular histones in mammalian systems activate innate immunity via TLR2/TLR4/TLR9-MyD88 signaling (de Vries *et al*. 2023), while their DNA-binding capacity modulates transcriptional activity to indirectly restrict viral replication (Li *et al*. 2022). Functioning as cytosolic viral dsDNA sensors, histones activate IFN-β pathways to inhibit DNA viruses including vaccinia virus, HPV-11/16, and adenovirus type 5 (AdV-5) (Kobiyama *et al*. 2010).

LD-resident immune proteins-including viperin, IRGM, and histones-construct a multilayered antiviral defense network through multidimensional mechanisms encompassing metabolic interference, signal amplification, and direct molecular interplay. Viperin exemplifies broad-spectrum antiviral activity via its enzymatic disruption of viral replication machinery, whereas IRGM orchestrates bidirectional autophagic regulation to contextually balance proviral and antiviral outcomes. Histones expand the conceptual framework of “metabolic immune” crosstalk through cross-domain defensive functions, leveraging their dual roles in chromatin biology and innate immunity to neutralize pathogens. This triad synergistically redefines host-pathogen conflict dynamics, demonstrating how spatial organization of immune effectors on LDs enables precision targeting of viral vulnerabilities while systemically reprogramming cellular metabolic-immune circuits.

## CONCLUSION AND FUTURE PROSPECTIVE

LDs, functioning as dynamic nexuses of cellular lipid metabolism and immune regulation, exhibit a “double-edged sword” duality in the host-virus arms race: they can be hijacked by viruses as resource depots while simultaneously serving as host-constructed metabolic immune fortresses (Fig. 1). As obligate intracellular parasites, viruses must plunder all resources from host cells. Viral infections typically induce profound LD phenotypic remodeling. Enveloped viruses predominantly drive LD clustering to procure neutral lipids for envelope biogenesis while securing energy platforms to fuel replication and dissemination, whereas non-enveloped viruses activate lipolytic/lipophagic programs to catabolize LDs into FFAs that energize viral factories. This universal “metabolic piracy” strategy exploits LD surface proteins as molecular anchors to couple viral replication complexes with LD-ER/mitochondria membrane contact sites, creating metabolically privileged microenvironments for efficient replication.

**Figure 1:**
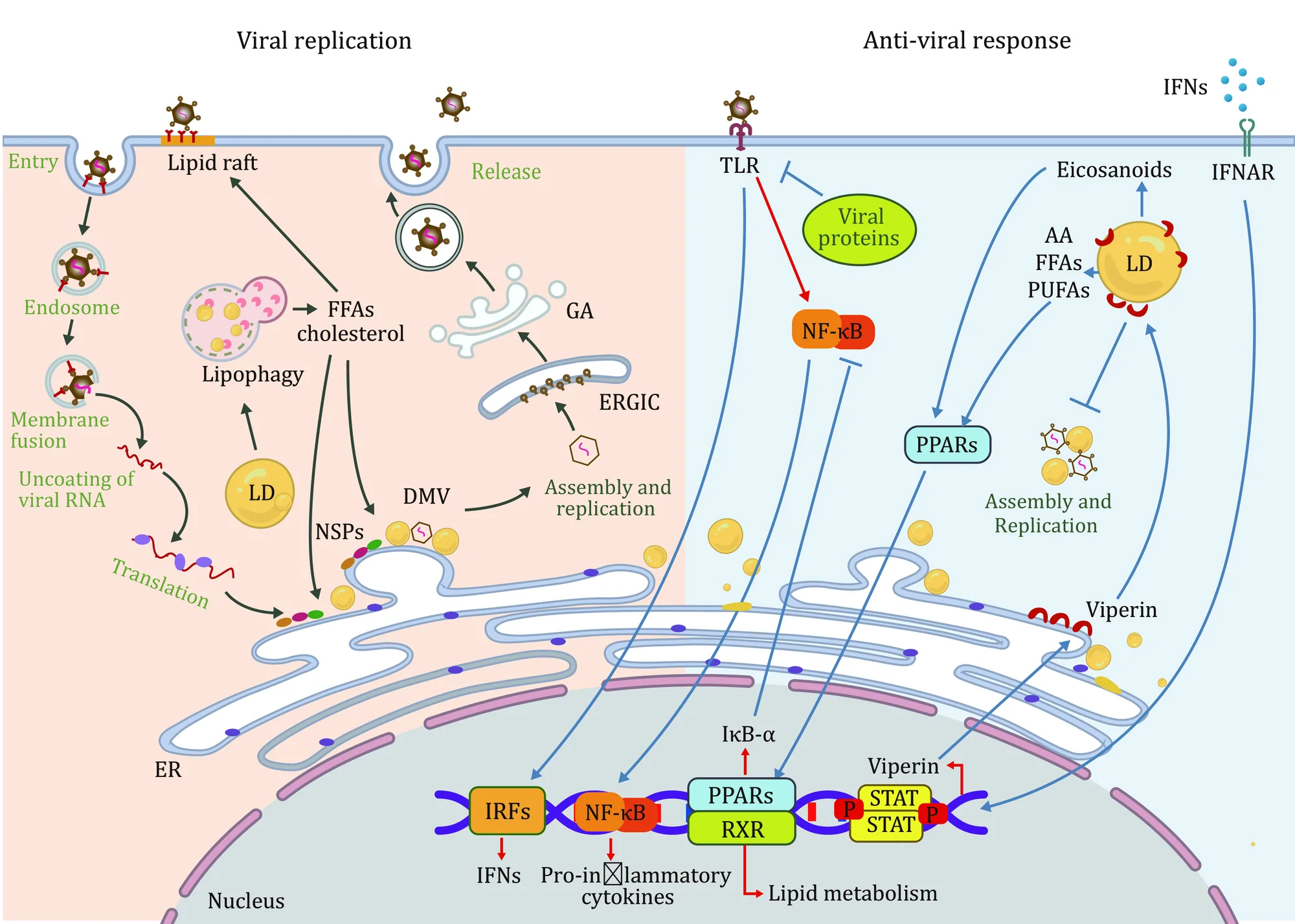
The dual roles of lipid droplets during viral infection. On one hand, viruses exploit LDs as platforms for the replication and assembly of viral particles, while simultaneously inducing lipolysis to release FFAs that energize these processes. On the other hand, the same viral entry activates TLRs, triggering an interferon response within the nucleus and stimulating LD-associated proteins to exert antiviral functions. At the same time, the metabolic byproducts of lipid droplets can act as agonists of PPARα/PPARγ, thereby inhibiting the pro-inflammatory response elicited by viral infection

However, evolutionary adaptation has endowed LDs with multilayered defensive mechanisms: (1) sequestering toxic lipids and viral components; (2) amplifying innate immune signaling cascades; (3) storing antimicrobial peptides and antioxidant molecules like vitamin E. Although the emerging field of LD Immunity has unveiled its potential as “metabolic immune interfaces”, critical questions remain unresolved: how do LDs dynamically balance proviral and antiviral functions? Does LD-mediated immunoregulation exhibit cell type-specific heterogeneity? Can targeted LD metabolic reprogramming selectively enhance host defense without disrupting metabolic homeostasis? Systematic deconvolution of the LD-virus-immune triad will enable development of precision “dual-edge modulation” strategies, paving the way for next-generation antiviral therapies through metabolic immune therapeutics.
